# Supplementary material for: Strain fingerprinting of exciton valley character in 2D semiconductors
Source: Nat Commun. 2024 Aug 30;15:7546. doi: 10.1038/s41467-024-51195-y (PMC11364664; doi:10.1038/s41467-024-51195-y)
Supplement: Supplementary file 1 — Supplementary Information File [file 41467_2024_51195_MOESM1_ESM.pdf]

Supplementary Information

Strain fingerprinting of exciton valley character in 2D semiconductors

Abhijeet M. Kumar[1,*], Denis Yagodkin[1,*], Roberto Rosati[2], Douglas J. Bock[1], Christoph Schattauer[3], Sarah Tobisch[3], Joakim Hagel[4], Bianca Höfer[1], Jan Kirchhof[1,5], Pablo Hernández López[6], Kenneth Burfeindt[1], Sebastian Heeg[6], Cornelius Gahl[1], Florian Libisch[3], Ermin Malic[2], and Kirill I. Bolotin[1,+]

[*] These authors contributed equally
[+] Corresponding author

[1]Department of Physics, Freie Universität Berlin, Arnimallee 14, 14195 Berlin, Germany
[2]Philipps-Universität Marburg, 35032 Marburg, Germany
[3]Institute for Theoretical Physics, TU Wien, Wiedner Hauptstraße 8-10, 1040 Vienna, Austria
[4]Department of Physics, Chalmers University of Technology, 41296 Gothenburg, Sweden
[5]Kavli Institute of Nanoscience, Department of Quantum Nanoscience, Delft University of Technology, 2628CJ Delft, The Netherlands
[6]Institute for Physics and IRIS Adlershof, Humboldt-Universität Berlin, Newtonstraße 15, 12489 Berlin, Germany

Note S1: Theoretical calculations for exciton strain response 2
Note S2: Theoretical calculations of D1 and D2 excitons 2
Note S3: Theoretical calculations of photoluminescence with KK-KQ hybridization 3
Note S4: Voltage to strain conversion in suspended devices 5
Note S5: Strain distribution in the sample 6
Note S6: Estimation of V_G-induced carrier density 6
Figure S1 Extended theoretical analysis of excitonic shifts and binding energy changes vs. strain. 8
Figure S2 Full range PL map in 1L-WSe_2 and 1L-WS_2. 9
Figure S3 Voltage to strain conversion in suspended devices. 10
Figure S4 Extended analysis of data in 1L-WSe_2. 11
Figure S5 Laser power dependence of excitons in WS_2 and WSe_2. 12
Figure S6 Estimation of strain inhomogeneity in a suspended device. 13
Figure S7 Extended analysis on KQ excitons in 1L-WSe_2 14
Figure S8 Strain-independent free exciton features in WS_2 15
Figure S9 PL vs. strain in a bilayer WSe_2 16
Figure S10 Detailed analysis of quantum-confined excitons in 1L-WSe_2. 17
Figure S11 Effect of strain inhomogeneity on KK-KQ hybridization PL. 18
Figure S12 Extended analysis of defect exciton orbital composition 19

Note S1: Theoretical calculations for exciton strain response

The strain-dependent excitonic energies in Fig. 1 of the main manuscript are obtained by investigating homogeneous lattice deformations. In particular, excitonic energies have been microscopically calculated by solving the Wannier equation with a generalized Rytova-Keldysh potential[1–3]. Here, we started from the unstrained single-particle dispersion relation[4]. We then include the strain-induced effective mass variations[5] and spectral shifts[6] (see Table 1). Note that we have taken a 10 meV smaller energy of the K valley in the conduction band to match the energy with the experiments and this is justified by the uncertainty in the energy separation between K and Q valleys[4] as well for the spin-orbit splitting[7,8]. The exciton energies in the bilayer case are also calculated with the generalized Wannier equation and then modified by diagonalizing a 4x4 matrix, taking into account the layer hybridization of the different exciton states[9].

Table 1: Adapted single-particle bandgap energies relative to the KK bandgap (cf. Ref. 4 with a 10 meV smaller KK energy), their strain-induced variation, and binding energy ($\Delta E_{binding}$) change per percentage of biaxial strain[6] for three different valleys in the WS_2 and WSe_2 monolayer

	Valley	Valley bandgap - KK bandgap (meV)	Spectral shifts (meV/%)	$\Delta E_{binding}$ (meV/%)
WS_2	KK	0	133.5	7.7
	KQ	37	-37	1.6
	ΓQ	306	83.5	5.6
WSe_2	KK	0	118.8	8.2
	KQ	5	-32.8	4.1
	ΓQ	511	61.8	23.8

The adapted parameters result in a very good agreement between theory and experiment, although a residual uncertainty is present due to the single-particle inputs[10,11]. While slightly different strain-induced shifts of the single-particle bandgap[5,6] would result in quantitative variations of the gauge factors, the different studies agree on the smaller gauge factor of ΓQ excitons compared to the KK excitons, in full agreement with the behaviour of the peak $X_{\Gamma Q}$ observed in our experiments. As reported in the main manuscript, most of the effective masses vary weakly with strain, with the exception of the one for the Γ valley in the valence band[5,12]. This results in almost strain-independent binding energies for all valleys, with relative variation of the order of 1.6% and 3% for KK and ΓQ excitons in WS_2 under 1% strain, cf. Fig. S1.

We stress that the relative position of ΓQ excitons in comparison to the bright KK excitons is drastically affected by the dielectric screening, as shown in Fig. S1(e). In particular, moving from free-standing to hBN-encapsulated samples, the energy separation $E_{\Gamma Q}$-E_{KK} increases by almost 50 meV in view of the binding energy variation, cf. Fig. S1(d) vs Fig. S1(e). Assuming that the photoluminescence is dominated by the thermalized distribution, we predict that at 10 K the relative intensity $X_{\Gamma Q}/X_{KK}$ of the ΓQ and KK peaks would be $e^{\frac{50\,meV}{k_B T}} \approx 10^{25}$ times smaller for the hBN-encapsulated samples in comparison to the free-standing ones. This indicates the crucial role played by our experimental apparatus allowing free-standing samples at cryogenic temperatures.

Note S2: Theoretical calculations of D1 and D2 excitons

We simulate the strain dependence of the D1 and D2 excitons using the Vienna Ab-Initio Simulation package (VASP). We set up a 6×6 supercell using a 300 eV energy cutoff, a PBE functional, a 3×3 k-point grid to distinguish localized from dispersive states, as well as 30 Å vacuum in z-direction. We remove a single chalcogen atom from the supercell (see Fig. S12). To assess the strain dependence, we biaxially and homogeneously stretch the supercell by up to 2%. All defect geometries are fully relaxed for each strain value using collinear spin-polarized calculations. Final energies are taken from non-collinear calculations for the relaxed geometry. We find that individual relaxation at each strain value is critical for accurate gauge factors, while further ionic relaxation using a non-collinear calculation does not significantly affect results (energy changes below 5 meV). As PBE underestimate the TMD band gap, we correct final energies by one constant for each material to fit to experimental values of the bulk band gap.

In the strain regime we consider, we find a linear shift of the ground state energy, as well as of individual Kohn-Sham eigenenergies. We find that the change in Kohn-Sham eigenenergies quantitatively fits to the experimentally

observed gauge factors, without accounting for the (smaller) strain dependence of the excitonic binding energy: Two pairs of localized defect states (spin degeneracies for each pair smaller than 3 meV) appear slightly below the CB minimum. All defect states are well localized around the defect site, with dispersions below 10 meV due to the finite supercell size.

Finally, we investigate the orbital character of the defect states, and their evolution with strain. We find all defect states to be dominated by the d-orbitals of the neighbouring three W atoms. Geometry optimization yields a Jahn-Teller distortion with two W-atoms featuring almost identical overlaps different from the third. While all five d orbitals are involved, dxy dominates for the paired W atoms, and dx^2-y^2 for the unpaired one. In contrast, the CB at the K-point is dominated by dz^2 orbital (unlike the Q-point where the orbital composition features a mix of different d-orbitals, Fig. S1(a)). The strain dependence of the orbital composition (see Fig. S12) is also linear, and quite small.

Note S3: Theoretical calculations of photoluminescence with KK-KQ hybridization

Here, we outline a model of strain-dependent KK-KQ hybridization. In order to model the experiments, we introduce a Gaussian distribution of exciton density, $N(\mathbf{r})$, and strain $s(\mathbf{r})$,

$$N(\mathbf{r}) = N_0 e^{-\frac{r^2}{2\Delta_r^2}} \quad \text{and} \quad s(\mathbf{r}) = s_0 e^{-\frac{r^2}{2\Delta_s^2}}, \tag{1}$$

with N_0 and s_0 being the maximum exciton density and strain, respectively. In the low exciton-density regime considered in our simulation, N_0 has no impact on the spectral shape of the photoluminescence, but only on the overall intensity. In contrast, the maximum strain s_0 crucially affects the optical response by changing the relative energy E_v^s of different excitonic valleys v, cf. Fig. S1. Here, we assume the width of the strain and excitonic profile Δ_s and Δ_r corresponding to a FWHM of 6.1 μm and 2 μm, respectively. Such a shape of $s(\mathbf{r})$ agrees well with the experimental strain profile in the center of the sample (cf. Fig. 3c in the main manuscript and Fig. S5), while we take an excitonic profile twice larger than the 1 μm laser spot, since the photoluminescence profile is typically larger than the laser width due to the exciton diffusion and related effects[13,14]. The photoluminescence spectrum $I(\mathbf{r}, E)$ becomes space-dependent due to the inhomogeneous excitonic distribution $N(\mathbf{r})$ and energies $E_v^{s(\mathbf{r})}$, with

$$I(\mathbf{r}, E) \propto N_{br}(\mathbf{r}) I_{s(\mathbf{r})}(E), \tag{2}$$

where $N_{br}(\mathbf{r})$ and $I_{s(r)}(E)$ are the bright-exciton density and strain-dependent emitted spectrum, respectively, and they read

$$N_{br}(\mathbf{r}) \equiv N_{br}\big(s(\mathbf{r})\big) \propto f_{br}\big(s(\mathbf{r})\big) N\big(s(\mathbf{r})\big) \equiv \frac{e^{-\frac{E_{\mathrm{KK}}^{s(r)}}{k_B T}}}{\sum_{\mathbf{Q},v} g_v e^{-\frac{E_{\mathrm{v}}^{s(r)} + \frac{\hbar^2 Q^2}{2M_v^{s(\mathrm{r})}}}{k_B T}}} N\big(s(\mathbf{r})\big) \quad \text{and}$$

$$I_{s(r)}(E) \propto \frac{\gamma\big(\gamma + \Gamma_{br}^{s(r)}\big) h_{s(r)}}{\big(E - E_{\mathrm{KK}}^{s(\mathrm{r})}\big)^2 + \big(\gamma + \Gamma_{br}^{s(r)}\big)^2} \tag{3}$$

The bright exciton density $N_{br}\big(s(\mathbf{r})\big)$ depends on the optically injected exciton density $N(\mathbf{r})$ time the fraction of bright ($Q = 0$ in KK valley) states $f_{br}(s)$, which assuming local equilibrium depend on strain as $f_{br}(s) = e^{-\frac{E_{\mathrm{KK}}^{s(r)}}{k_B T}} / \sum_{\mathbf{Q},v} g_v e^{-\frac{E_{\mathrm{v}}^{s(r)} + \frac{\hbar^2 Q^2}{2M_v^{s(\mathrm{r})}}}{k_B T}}$, where g_v, M_v and T are the valley degeneracy, the valley total mass and the temperature, respectively. The spectrum $I_s(E)$ describes the PL emitted around energy $E_{\mathrm{KK}}^{s(\mathbf{r})}$ via an Elliott formula[10] with the radiative-recombination rate γ (for WSe_2 we use γ =1.15 meV[5]) and the exciton-phonon scattering rate $\Gamma_{br}^{s(r)}$ of bright excitons , which we evaluate microscopically in the Born-Markov approximation[15,16] starting from the strain-dependent exciton energies (see Note S1) and the DFT inputs for phonon energies and electron-phonon scattering [17]. Finally, $h_{s(r)}$ describes the strain-dependent exciton hybridization. In the uncoupled case, KK and KQ excitons are represented by $|\mathrm{KK}\rangle$ and the 3-fold degenerate state $|\mathrm{KQ}\rangle$, respectively. While a fully microscopic description of the hybridization process goes beyond the scope of the present work, here we

assume that after the coupling the eigenstates are given by $|a\rangle = \sqrt{1 - A^2_{s(r)}}|\text{KK}\rangle + \sqrt{A^2_{s(r)}}|\text{KQ}\rangle$ and the three-fold degenerate state by $|b\rangle = -\sqrt{A^2_{s(r)}}|\text{KK}\rangle + \sqrt{1 - A^2_{s(r)}}|\text{KQ}\rangle$ with

$$A^2_{s(r)} = A^2_0 e^{-\frac{\left(E^{s(r)}_{\text{KK}} - E^{s(r)}_{\text{KQ}}\right)^2}{2\Delta^2_E}} \quad , \tag{4}$$

where A_0 corresponds to the maximum mixing. Equation (4) implies that the mixing between $|\text{KK}\rangle$ and $|\text{KQ}\rangle$ takes place only when KK and KQ become quasi-degenerate thanks to strain, i.e. $E^{s(r)}_{KK} \approx E^{s(r)}_{KQ}$, whereas in the opposite limit of $\left|E^{s(r)}_{KK} - E^{s(r)}_{KQ}\right| \gg \Delta_E$ one has $A_{s(r)} \approx 0$, recovering the uncoupled regime $|a\rangle \approx |\text{KK}\rangle$ and $|b\rangle \approx |\text{KQ}\rangle$. In view of the three-fold degeneracy of $|\text{KQ}\rangle$, the hybridization increases the occupation of KK states to $h_{s(r)} = 1 + 2A^2_{s(r)} > 1$, again recovering the uncoupled occupation of $h_{s(r)} = 1$ for $A^2_{s(r)} \to 0$ (regular emission from KK without contributions from the hybridization). Here and in the main paper we take $A^2_0 = 0.5$ and Δ_E corresponding to a FWHM of 10 meV in the Gaussian in Eq. (4).
Finally, the space-integrated PL is given by

$$I_{2D}(E) = \int dxdy\, I(\mathbf{r}, E) \equiv \int dr I^{\{r\}}_{2D}(E), \;\text{ with }\; I^{\{r\}}_{2D}(E) = 2\pi r\, N_{br}(r) I_{s(r)}(E)\,, \tag{5}$$

where we made use of the angular symmetry of both the exciton and the strain profile. We stress that while the density of excitons ($N_{br}(r)$) peaks at the centre of the membrane, the contribution of the region away from the center is non-negligible due to the factor $2\pi r$ in Eq.(5).

In our calculation we consider the strain profile mentioned in the main text (Eq.(1) with FWHM of 6.1 μm and maximum $s_0 = 0.5\%$, Fig. S11(a)), which results in the angle-integrated PL: $\text{I} \equiv \text{I}^{\{r\}}_{2\text{D}}(\text{E})$ (Eq. (5)) shown in Fig. S11(b). It presents energy-symmetric peaks centered at the bright-exciton energy $\text{E}^{\text{s(r)}}_{\text{KK}}$, this energy depending on the strain at the specific distance (increasing by 15 meV in the first 2 μm, cf. the red dashed line). The KQ energy $E^{s(\text{r})}_{\text{KQ}}$ (blue) is almost degenerate with E_{KK} at r ≈ 2 μm, where the space-resolved contribution $I^{\{r\}}_{2D}(E)$ is still finite thanks to the radial factor, cf. Eq. (5).

The space-integrated photoluminescence $I(E)$ has asymmetric spectrum (Fig. S11(e)), showing a high-energy tail both with and without the KK-KQ coupling (blue and red line, respectively). This asymmetry is induced by the PL emitted at larger distances from the center, where the emission energy $\text{E}^{\text{s(r)}}_{\text{KK}}$ is higher, cf. Fig. S11(b). Here we have not included any extrinsic inhomogeneous broadening of exciton spectrum, which could potentially decrease the asymmetry while increasing the linewidth. In addition, including the KK-KQ hybridization we find the new shoulder (blue line in Fig. S11(e)). The high-energy emission from the sides of the excitonic distribution also affects the linewidth, which we show in Fig. S11(e) as a function of the maximum strain s_0 as well as by varying the strain inhomogeneity (yellow to dark orange lines). For strain much broader in space than the exciton distribution (dark orange), the linewidth is flat with s_0, except for two reductions of the linewidth at the specific strain values of $s_0 \approx 0.15\%$ and $s_0 \approx 0.25\%$ (grey lines vertical lines in Fig. S11(e)). This reflects the closing of the scattering channel from KK to KQ states, when the strain-dependent energy separation between the two excitons is smaller than the energy of the involved intervalley optical and acoustic phonons (taken from DFT studies [17]), respectively. For strain profiles comparable with the exciton peak (yellow line in Fig. S11(e)) we find an increase of the linewidth with s_0 that is induced again by the spatial integration over regions with PL emitting at different energies. In addition, we can distinguish the linewidth reductions at $s_0 \approx 0.15\%$ and $s_0 \approx 0.25\%$ due to the closing of the KK to KQ scattering channel as well as the linewidth increase at $s_0 \approx 0.4\%$ induced by the exciton hybridization.
Finally, in Fig. S7(a,b) we show the theoretically predicted space-integrated PL both with and without KK-KQ hybridization (blue and red) for three values of maximum strain (Fig. S7(a)) as well as the corresponding difference between the predictions with and without hybridization (Fig. S7(b)). In both cases, we find the main peak X^0_{KK} red-shifting with increasing strain s_0, while a new peak appears for the strain values of $s_0 \gtrsim 0.4\%$, first overlapping with the KK peak for $s_0 \approx 0.45\%$ and then creating a separate peak for $s_0 \gtrsim 0.5\%$ (Fig. S7(a)). Considering the difference between the cases with and without hybridization (Fig. S7(b)), we show how the hybridization provides a new contribution always at the given energy peak $E \approx E^{s(\text{r})}_{\text{KK}} \approx E^{s(\text{r})}_{\text{KQ}}$ and with decreasing intensity for $s_0 \gg 0.5\%$.

Note S4: Voltage to strain conversion in suspended devices

To quantitatively determine strain in our devices, we use laser interferometry measurements. In general, our device structure can be considered as an optical cavity consisting of the suspended flake and the SiO_2/Si underneath. The change in the intensity of the laser light reflected from the cavity vs. membrane deflection can described as:

$$\Delta I_{laser} \sim A(V_G)\, sin^2\left(4\pi \cdot \frac{d(V_G)}{\lambda} + \varphi\right) \tag{7}$$

where $d(V_G)$ is the deflection of the flake due to an applied gate voltage, φ is the initial phase and λ is the wavelength of the laser. The term $A(V_G)$ describes changes in the optical constants of suspended 2D material under strain. Since the laser energy used in our experiments is ~100 and ~220 meV away from the excitonic resonance in WSe_2 and WS_2, respectively, a strain-induced modulation in the excitonic band structure may slightly change the optical constants. Therefore, we assume the pre-factor in Eq. 7 to be V_G dependent, and approximate this as $A(V_G) = A_0 + A_1 V_G^2$ via a Taylor expansion. Note that there is no linear term in the expansion since the force acting on the flake – and hence all optical constants – are even functions of V_G.

The deflection of the flake directly relates to strain via a simple approximation[18]:

$$d(V_G) = R\sqrt{1.25\varepsilon(V_G)} \tag{8}$$

, where $\varepsilon(V_G)$ is the applied strain, R is radius of the membrane, and the factor 1.25 is corresponds to the approximation of a circular shape[19,20]. We note that strain from Eq. 8 is close (less than 5% difference) to a more precise approach in Ref.[21].

In principle, the strain can be calculated by fitting Eq. 7 to experimentally obtained interferometry data (Fig. S3). However, the accuracy of the extraction can be increased by analyzing photoluminescence vs. V_G data obtained in the same conditions as the interferometry measurements. We follow a well-accepted[12,22–26] assumption that the energy shift of KK excitons ΔE linearly depends on strain ε:

$$\Delta E(V_G) = \Omega_{\mathrm{KK}} \cdot \varepsilon(V_G) \tag{9}$$

, where Ω_{KK} is the strain gauge factor. Finally, we combine the equations above:

$$\Delta I_{laser}(V_G) = (A_0 + A_1 V_G^2)\, sin^2\left(4\pi R \cdot \frac{\sqrt{1.25\left(\frac{\Omega_{\mathrm{KK}}}{\Delta E(V_G)}\right)}}{\lambda} + \varphi\right) \tag{10}$$

We fit this expression to experimentally obtained $I_{laser}(V_G)$ data (Fig. S3) treating gauge factor Ω_{KK}, initial strain ε_0, and amplitudes A_0, A_1 as the free parameters and using $\Delta E(V_G)$ from photoluminescence measurements. Since the shift of the peak position at low V_G brings large uncertainty as $1/\Delta E(V_G) \to +\infty$ in Eq. 10, we reduce the contribution of these points in the fit using weight function $w(V_G) = \left|\frac{1}{1+e^{(V_G - V_{th})/s}} - \frac{1}{1+e^{-(V_G - V_{th})/s}}\right|$, where V_{th} is threshold voltage (35 V for WSe_2 and 10 V for WS_2) and parameter $s = 5$ V controls smoothness.
We obtain 102±13 meV/% of KK excitons in WS_2 and 112±6 meV/% for KK in WSe_2, close to the values previously reported at room temperature[27] and consistent with calculations (Fig. S1). Having experimentally determined the gauge factor for KK excitons, we can use their spectral position as strain indicator.

Note S5: Strain distribution in the sample

The device homogeneity plays crucial role in our experiments via limiting peak broadening, influencing correct assessment of strain gauge factors, and fine-tuning the hybridization of specific excitons. In this note, we examine the uniformity of pre-strain in our devices, effects of heating, and the fitting procedures considering the strain distribution and quality of the sample.

Device uniformity. Device uniformity is influenced by i) pre-strain, and ii) spatial strain-gradient under high gate voltages.

To assess device uniformity and pre-strain consistency, we measured spatial PL maps at room temperature (Fig. S6a). Our analysis of the spatial variation in the excitonic energy confirms a small strain inhomogeneity of 0.03% in the pristine device. The same observation was recorded when the device was pumped down (Fig. S6b), confirming a uniformity in the pre-strain. Additionally, we insured device stability against any pocket of air trapped air inside the cavity by implementing a vent channel for pressure equilibration (see Methods in the main text).

The strain inhomogeneity increases under the application of large gate voltages. Using a combination of COMSOL simulation and spatial mapping of neutral exciton peak, we find that within 1 μm from the center of the membrane, the strain decreases to $0.9{\cdot}\varepsilon_{center}$ (Fig. S6d,e). Note, that the degree of homogeneity can be controlled by controlling the size of the circular trench. To optimize our experiments, we ensure that the laser spot (diameter ~1 μm) is tightly-focused in the center of the membrane.

Heating effects. The strain response in our devices may be affected by heating effects leading to undesirable thermal expansion or contraction. Heating can arise from either the application of large gate voltages or laser absorption by the membrane. The former effect is negligible since the current generated in our capacitor-like device stays below 10 nA even for a gate voltage exceeding 150 V that dissipates over the entire area of the conductive pad. In contrast, laser absorption in the center of the membrane locally increases the sample temperature. This, in turn, relaxes the net strain in the membrane due to a positive thermal expansion coefficient of the TMDs[28]. For a precise estimation, we simulate the laser heating-induced strain profile in a monolayer WSe_2 device at T = 5 K (Fig. S6c) and find the strain variation to be on the order of the pre-strain in our device.

In our study, we approximate the laser heating effect as a static strain component comparable to the pre-strain. Although the cavity interference effects and the band structure modulation may change the absorbed laser power, the related strain magnitude is nearly constant and remains more than an order magnitude smaller compared to the maximum applied strain via electrostatic pressure.

Broad excitonic linewidth and fitting error. Identification of peaks is hampered by peak broadening due to pre-strain variation, membrane heating, and, most importantly, spatial inhomogeneity of the applied strain (see Fig. S11). We note that the excitonic peaks in our devices are broader compared to state-of-the-art hBN-encapsulated devices. Additionally, some features are weak in intensity and only appear at specific strain values. Therefore, to precisely determine the emission energy of excitons, a careful fitting procedure is necessary.

To this end, we use the second derivative of the PL intensity, d^2PL/dE^2, to obtain an initial guess for the PL peak positions from $E_{center} = \max(d^2PL/dE^2)$ and amplitude $A = PL(E_{center})$. We then fit the raw PL(E) spectra with several Gaussians using the initial guesses obtained above. We perform batch fitting to minimize selective biases during the fitting procedure. Finally, we estimate uncertainties (shadows in Fig. 2c,d) by varying the constraints/parameters of the fit (e.g. number of peaks used in fitting, peak-width, ascending and descending order of strains during fitting etc).

Note S6: Estimation of V_G-induced carrier density

The change in the carrier density ($n_{e,h}$) induced by the gate voltage in the center of the membrane can be estimated using a plate capacitor model:

$$n_{e,h} = \frac{(V_G - V_0)\epsilon_0}{e}\left(\frac{\epsilon_{SiO2}}{d_{SiO2} + \epsilon_{SiO2}(d_{Au} - d(V_G))}\right) \quad (11)$$

Here ϵ_0 and $\epsilon_{SiO2} = 3.6$ are the vacuum permittivity and the dielectric constant of SiO_2, respectively. $V_0 =$ -55 V is the gate voltage at which the sample is charge neutral. $d_{SiO2} =$ 900 nm is the SiO_2 thickness, $d_{Au} =$ 600 nm is the distance between the gold surface and SiO_2, $d(V_G)$ is the flake deflection (obtained from interferometry) and e is the elemental charge. From this method, we obtain $n_{e,h} \sim 0.8 \cdot 10^{12}$ cm^{-2} at $V_G =$ -150 V.

However, the accuracy of this method is significantly challenged by artefacts due to Schottky barriers, defects, photo-doping, etc., resulting into a nonlinear dependence of $\Delta n_{e,h}$ on V_G. We note that the experimental features of excitons and trions (intensity, energy separation, linewidth, etc.) carry an accurate measure of Fermi energy level shift, regardless of the artefacts present in the device. This exciton-trion energy separation (ΔE_{XT}) relates to the Fermi energy shift, as $\Delta E_{XT} \approx E_F$[29]. The carrier density, in turn, relates to the Fermi energy as

$$n_{e,h} = \frac{E_F m_{e,h}}{\pi h^2} \quad (12)$$

Here, $m_{e(h)} = 0.36\ (0.40) \cdot m_0$ is the effective mass of an electron (hole)[4]. We used Eq. S12 to estimate the carrier density and plotted in Fig. S4d. We find $n_h \sim 1 \cdot 10^{12} cm^{-2}$ at 1% strain. Overall, the maximum induced carrier density does not exceed $1.5 \cdot 10^{12}\ cm^{-2}$. We find that the estimated carrier density may vary within 30% across different approaches (such as capacitor model or trion/Fermi-polaron approximation in Eq. S12).

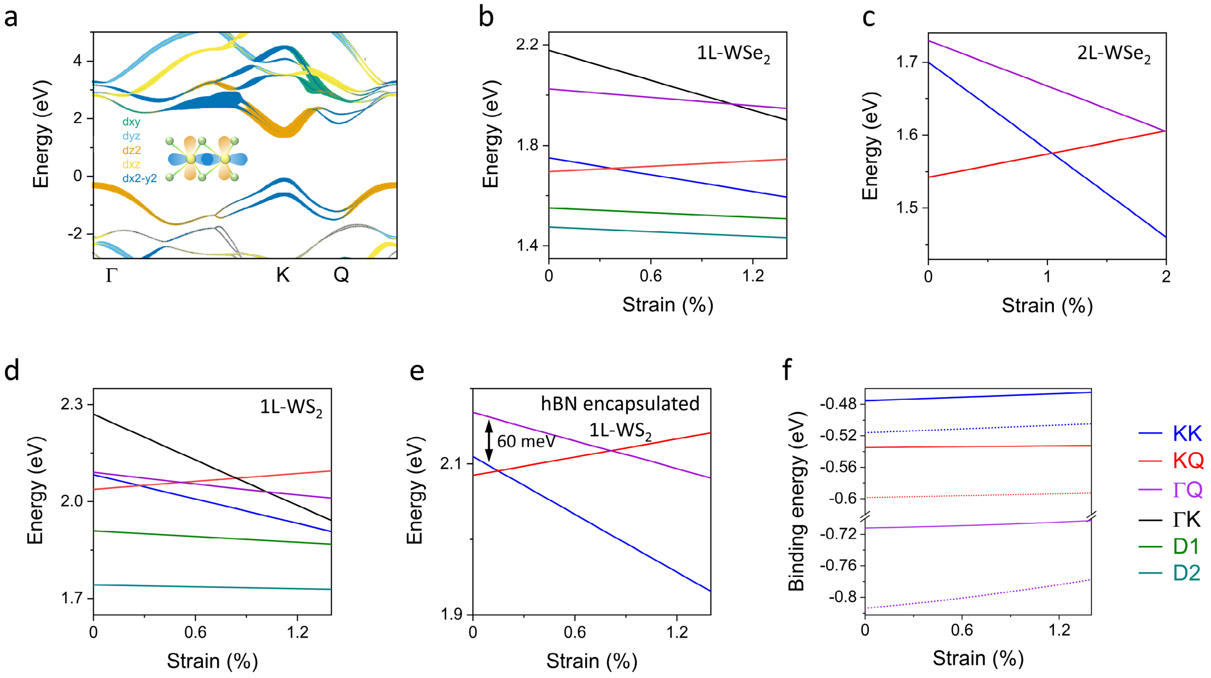

Figure S1 Extended theoretical analysis of excitonic shifts and binding energy changes vs. strain.

a) 1L-WSe_2 band structure (non-collinear) from VASP, without correction of the band gap. The dominant orbital contribution from the d-orbitals of W-atom are color-coded (see Fig. S12 for complete orbital weight). The dz^2 orbital dominates at the K-point in the CB and the Γ-point in the VB. Overlap of these orbitals of neighboring atoms is weakly dependent on strain (schematic in the inset). However, the K-point in the VB and the Q-point in the CB are prevailed by dx^2-y^2 orbitals, overlap of which significantly changes under strain (schematic in the inset). The apparent opposite strain responses of these valleys in calculations arise since we fix the K-point in VB and evaluate all other valleys relative to it.

b-e) Strain dependent energy for excitons in 1L-WSe_2, 2L-WSe_2, 1L-WS_2, and hBN encapsulated 1L-WS_2 calculated via solving the Wannier equation starting from DFT single-particle inputs (see SI Note 1): KK (blue), KQ (red), ΓQ (purple), ΓK (black). Many of these excitons are observed in our experiments. Some others we do not see. For example, ΓQ and ΓK excitons in an unstrained 1L-WSe_2 lie ~270 and ~420 meV above KK exciton and hence are not resolved in our PL experiments. A ΓK exciton in 1L-WS_2 lies lower but is still ~190 meV above KK, hence, is not optically active. We note that ΓQ exciton in a supported 1L-WS_2 has significantly higher energy compared to the suspended 1L-WS_2 that may drastically reduce its emission (see SI Note 1). D1 (green) and D2 (teal) are the two defect excitons originating from single chalcogen vacancies (see Note S3 for calculation details). Based on their relative energy positions, we assign the D^0 peak in Fig. 2 of the main text to D1 in 1L-WSe_2 and D2 in 1L-WS_2.

f) Binding energy vs. strain for excitons in 1L-WSe_2 (solid lines) and 1L-WS_2 (dotted lines). A weak (<10 meV/%) dependence of binding energy change on strain for KK and KQ excitons is consistent with the data in Fig. 2 of the main text. Meanwhile, ΓQ and ΓK experience around 20 meV/% change of binding energy thanks to larger strain-induced variations of the effective mass at the Γ-point.

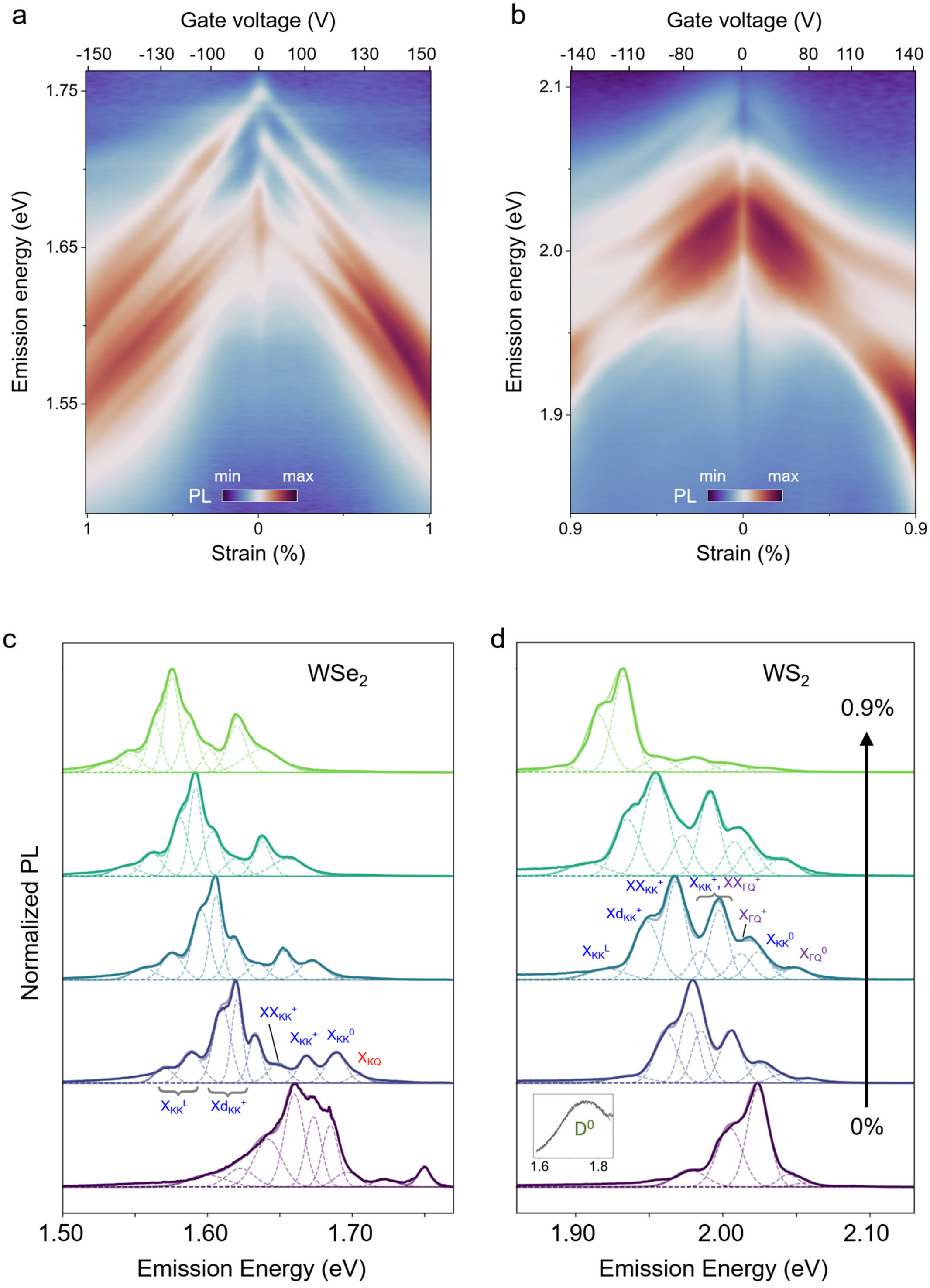

Figure S2 Full range PL map in 1L-WSe_2 and 1L-WS_2.

False color PL map for both polarity of V_G in **a)** 1L-WSe_2 and **b)** 1L-WS_2, plotted on a log scale. The strain response is symmetric with respect to the V_G polarity. The data in Fig. 2 of the main text is the strain response for negative V_G (p-doping) for both WSe_2 and WS_2. **c-d)** Selected PL line cuts from WSe_2 and WS_2 corresponding to the data in Fig. 2 in the main text. Dashed lines highlight individual peaks obtained from fits; cumulative fits are denoted by semi-transparent lines. Inset of Fig. d shows the D^0 in WS2, that appears near 1.76 eV and remains nearly strain independent. Note, that some low energy KK excitons are doping-dependent, and are prominent only in low strain regime. This serves as additional source of uncertainty in fitting procedure. In our analysis, we considered features that persisted for the entire range of V_G (Fig. 2 in the main text).

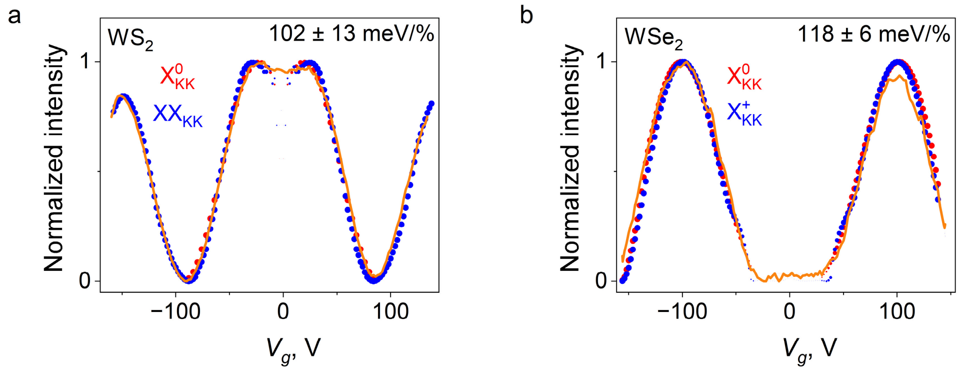

Figure S3 Voltage to strain conversion in suspended devices.

a) Normalized reflectance I_{laser} vs. V_G (orange line) in 1L-WS_2. Blue and red points are the fitted $I(V_G)$ using the procedure from Note S4, corresponding to $\Delta E(V_G)$ for $X_{KK}{}^0$ and $XX_{KK}{}^+$ excitons, respectively. The point size shows the weight in the fitting. The fit for $XX_{KK}{}^+$ yield the gauge factors of 100 meV/%. An average gauge factor from fits with $\Delta E(V_G)$ from different KK excitons yields Ω_{KK} =102$\pm$13 meV/% The uncertainty is dominated by variations in $\Delta E(V_G)$ for different excitons.

b) Normalized reflectance I_{laser} vs. V_G (orange line) in 1L-WSe_2 is fitted using the procedure from Note S4, with blue and red points corresponding to $\Delta E(V_G)$ for $X_{KK}{}^0$ and $X_{KK}{}^+$ excitons, respectively. The point size shows the weight in the fitting. The fit averaged over KK excitons yields Ω_{KK} = 118 $\pm$ 6 meV/%.

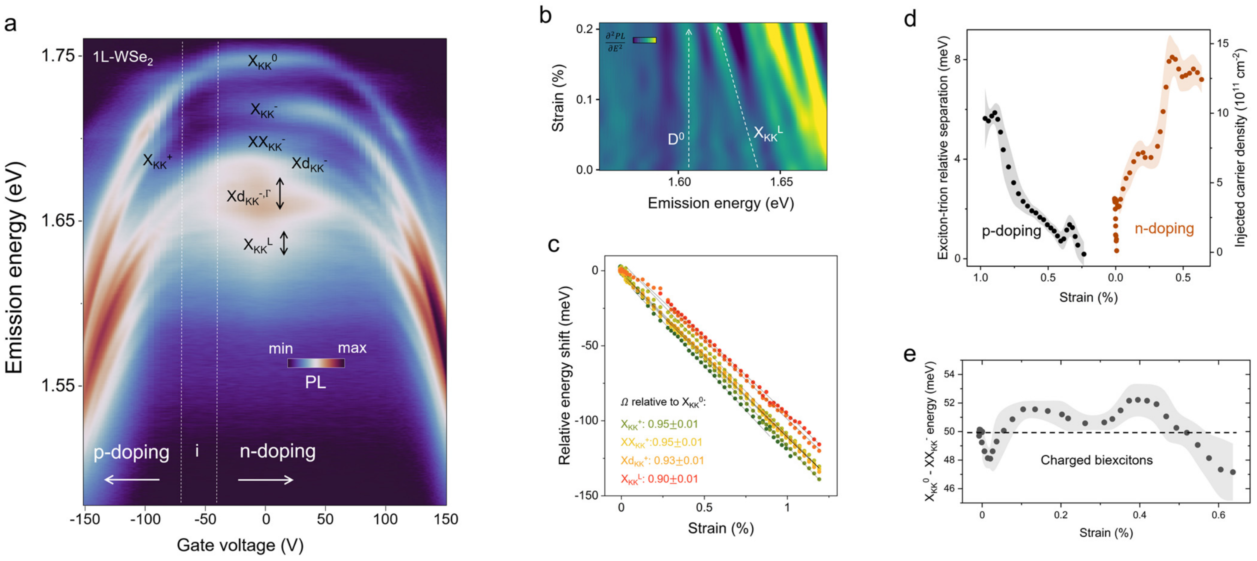

Figure S4 Extended analysis of data in 1L-WSe_2.

a) PL vs. V_G false color map for 1L-WSe_2 in the same device as in Fig. 2 of the main text. The doping regimes corresponding to p-doping, intrinsic (i) and n-doping are highlighted by dashed lines and arrows. Intrinsic region is characterized by the lowest intensity of the trion peak with respect to the neutral exciton intensity.

b) False color map of d^2PL/dE^2 vs. strain from the same device as in **a**. We see that the feature near 1.60 eV is nearly strain-independent compared to the peak near 1.635 eV. We identify these features as D^0 and X_{KK}^L, respectively. Note, that the spectral lines in our devices are broader compared to hBN-encapsulated samples. Because of that, certain states such as D^0 and X_{KK}^L are not immediately distinguishable.

c) Relative energy shift ($E(\varepsilon)$ - $E(\varepsilon = 0)$) vs. strain for various excitons in 1L-WSe_2 (corresponding to Fig. 2a,c in the main text). The energy shift for each state is fitted to a linear function and the Ω (relative to the X_{KK}^0) is extracted by comparing their slopes. We find that most of the KK excitons including trions, biexcitons, dark trions and their phonon replicas show very similar gauge factors with a variation less than 10%. This suggests that the effects related to doping changes, strain-dependent phonon energy and strain-dependent effective mass changes are below the uncertainty in our experimental data. A slightly lower Ω of X_{KK}^L ($\sim 0.9 \cdot \Omega_{XKK0}$) likely suggests a complex picture of exciton binding energy when the defects are involved. However, we note that the binding energy changes for these excitons are theoretically estimated to be below 20% of the gauge factor.

d) Changes of energy separation between the neutral and charged excitons vs. strain in 1L-WSe_2. Black and orange data points correspond to p-doped ($V_G < 0$) and n-doped ($V_G > 0$) regimes, respectively; the shadows denote error bar. We use relative energy separation between the neutral and charged excitons to estimate injected carrier (electron/hole) density[28,29] (see Note S6), plotted on the right Y-scale. We find the injected carrier density to be ~1.0 x 10^{12} cm^{-2} under 1% of applied strain (p-doped case, data in Fig. 2 of the main text). We find a variation in the induced carrier density by up to 30% across different approaches.

f) Energy separation between the neutral exciton (X_{KK}^0) and negatively-charged biexciton (XX_{KK}^-) vs. strain. There is no apparent dependence on strain although the variation is approximately 10%. The lack of strain dependence of the binding energy (< 5 meV/%) is consistent with the theoretical prediction for KK exciton effective mass (calculations in Fig. S1).

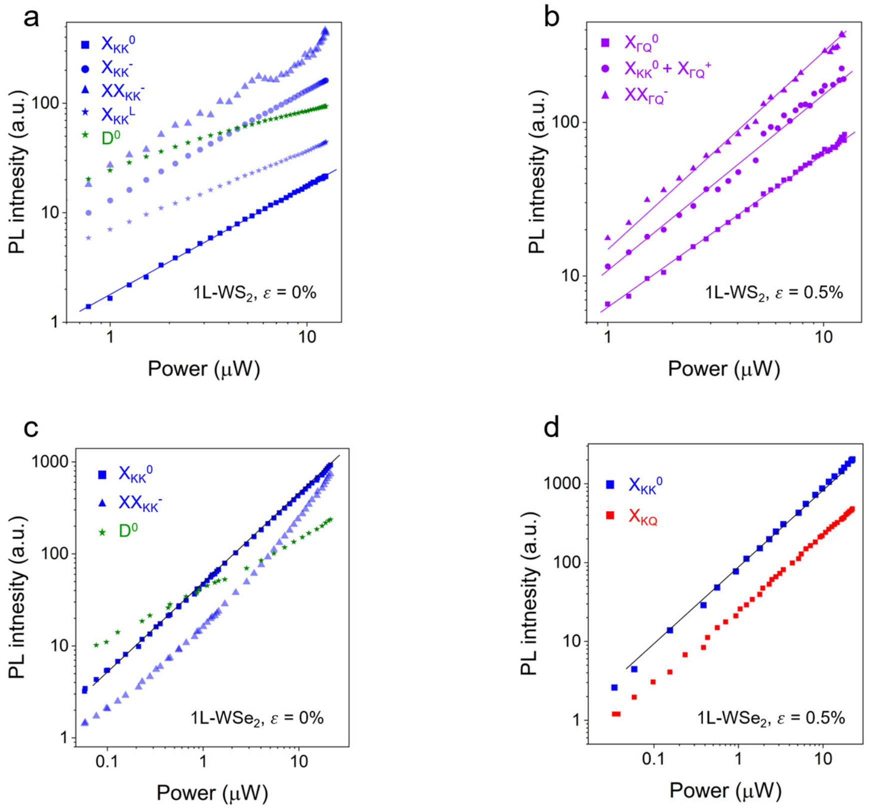

e

	X_{KK}^0	X_{KK}^+	X_{KK}^-	XX_{KK}^-	Xd_{KK}^-	$Xd_{KK}^{+/-}$ phonon replica	X_{KK}^L	D^0	$X_{\Gamma Q}^0$	X_{KK}^+ + $X_{\Gamma Q}^+$	$XX_{\Gamma Q}^+$	X_{KQ}	Y
WSe_2	0.97		1.15	1.42	0.9	0.81	0.70	0.66				0.95	
WS_2	0.97		0.99	1.33	0.97	0.94	0.86	0.50	1.01	1.18	1.36		1.05

Figure S5 Laser power dependence of excitons in WS_2 and WSe_2.

a,b) Laser power dependence of PL peaks of various KK and ΓQ valley excitons in 1L-WS_2. While most of the KK excitons can be identified in an unstrained device (V_G = 0 V), the ΓQ excitons are better resolved in a strained device ($\varepsilon \approx 0.5\%$).

c,d) Laser power dependence of KK and KQ excitons in 1L-WSe_2 (device 2). Similar to the ΓQ excitons in 1L-WS_2, X_{KQ} excitons in 1L-WSe_2 can only be resolved when strained ($\varepsilon \approx 0.5\%$).

e) Power exponent (α) for the various excitons in 1L-WS_2 and 1L-WSe_2 (a-d) extracted via fitting to a power law ($PL \propto P^{\alpha}$) where P is laser power. Our key observation is that $X_{\Gamma Q}^0$ and X_{KQ} both show linear power dependence. At the same time, a clear power dependence of $X_{\Gamma K}^+$ is hard to achieve as it is mixed with X_{KK}^0. We suggest that $XX_{\Gamma Q}^+$ is a biexciton like state since it shows super linear power dependence similar to XX_{KK}^- in 1L-WSe_2. Finally, we note that laser-induced heating effects are pronounced in a suspended membrane compared to supported devices. At high power (>10 μW), these effects cause strain changes, excitonic red shift and non-linear responses that, in turn, may slightly suppress the super-linear behaviour.

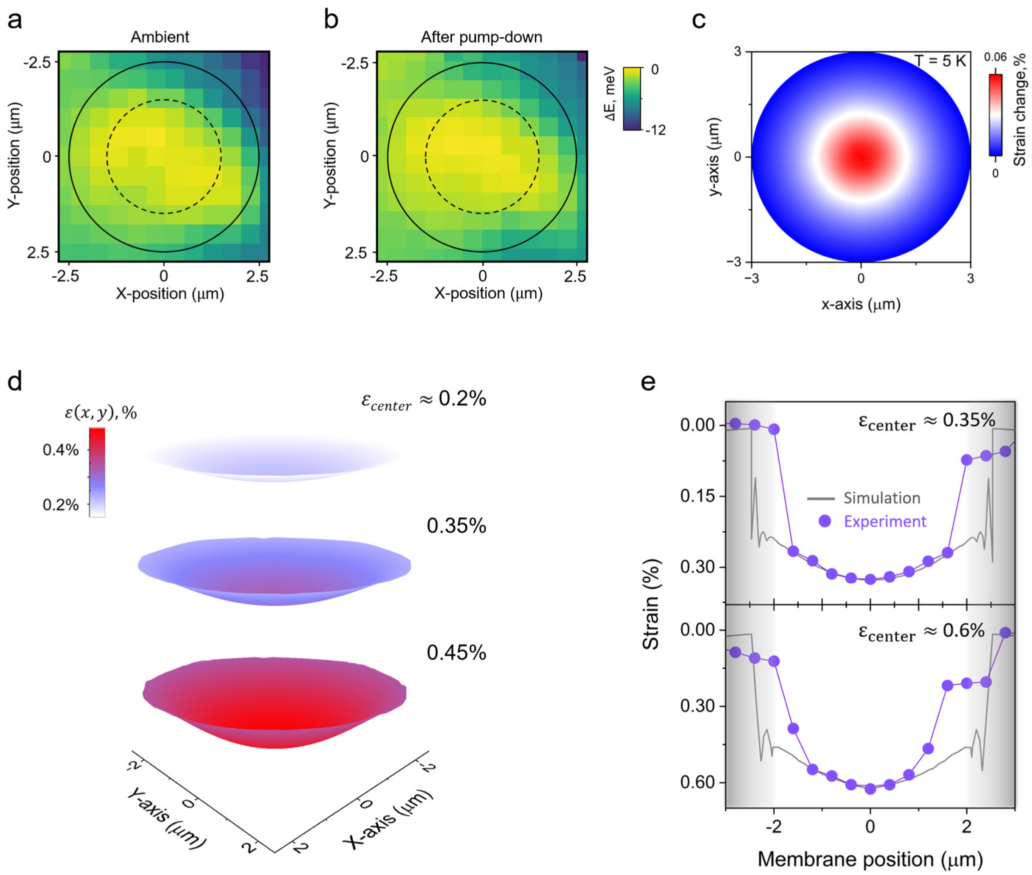

Figure S6 Estimation of strain inhomogeneity in a suspended device.

a,b) Spatial map of relative exciton energy shift in ambient (a) and after pump down (b), see Note S5 for details. The solid circle marks the edge of the trench, and the dashed circle indicates the region of 3 μm dimeter around the center of the membrane, considered as homogeneous region. Our analysis of the spatial variation in the excitonic energy confirms a small strain inhomogeneity below 0.05% in the pristine device.

c) Laser heating-induced change in strain in a suspended sample at 5 K, simulated via COMSOL (Note S5). We assume 10% absorption of the laser at 10 uW and with spot diameter of 1 μm. The heating effect is maximum in the center of the membrane and remains nearly uniform within ± 1 μm region. Assuming a positive thermal expansion coefficient, the laser heating relaxes the pre-strain. The laser heating is treated as a static effect, and the induced changes in strain remains more than an order of magnitude compared to the maximum applied strain.

d) COMSOL simulated biaxial strain profile ($\varepsilon_{xx} + \varepsilon_{yy}$) in a suspended WSe_2 membrane. The three panels (top to bottom) correspond to 0.2, 0.35, and 0.45% of applied strain values (ε_{center}) in the center of the membrane.

e) Estimation of spatial strain variation in a suspended WSe_2 device. The solid grey lines are strain value cuts across the membrane obtained from the simulations for two values of ε_{center}: 0.35% (top), 0.60% (bottom); spikes at the edges are due to numerical artefacts. The purple data points are the local strain values estimated from the X_{KK}^0 energy shift under simultaneous position-dependent excitation/probe. We find that within ± 1 µm from the center, the strain decreases to $0.9 \cdot \varepsilon_{center}$, consistent with the simulations. Near the membrane edges (grey shadow), the strain estimation is challenging due to mixed signal from the supported and suspended regions of the flake, as well as an increased strain nonuniformity. To avoid numerical artefacts, we approximate the strain profile near the edges (2 µm < |x| < 2.5 µm) with a parabolic extrapolation of the strain profile in the region |x| < 2 µm, also sketched in Fig. 3c of the main text.

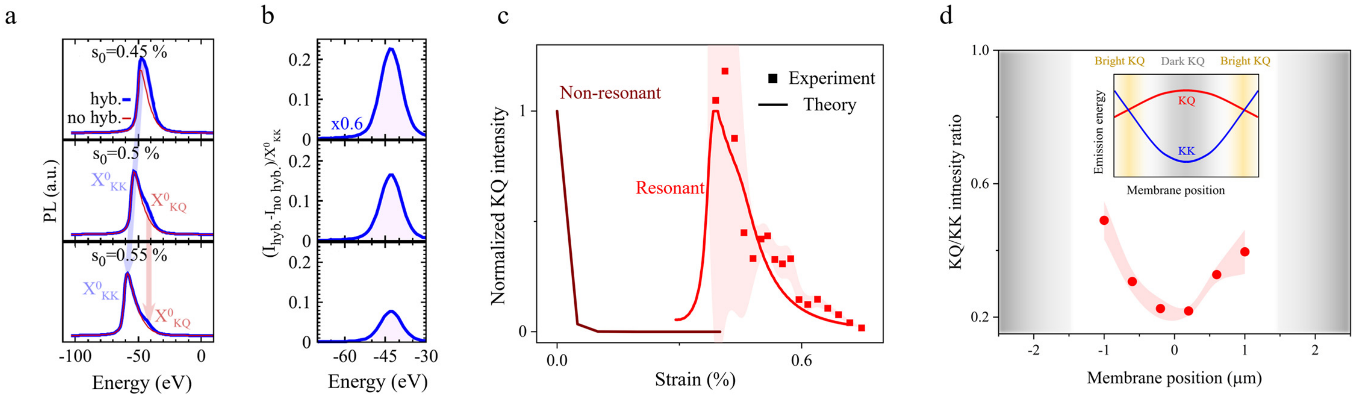

Figure S7 Extended analysis on KQ excitons in 1L-WSe₂.

a) Calculated space-integrated PL in 1L-WSe_2 for three values of maximum strain (0.45, 0.50 and 0.55 %). Blue and red lines represent the intensity with and without KK-KQ hybridization (I_{hyb} and $I_{no\ hyb}$, respectively). While the peak X_{KK}^0 red shifts with increasing strain s_0, the hybridization-induced peak is fixed at the given energy E for which $E \approx E_{\mathrm{KK}}^s \approx E_{\mathrm{KQ}}^s$.

b) The intensity difference between I_{hyb} and $I_{no\ hyb}$ relative to the X_{KK}^0 peak corresponding to the strain values in (a). The intensity of the hybridized peak relative to X_{KK}^0 decreases continuously for $s_0 > 0.4\%$.

c) Normalized KQ emission intensity vs. strain in 1L-WSe_2. The calculated non-resonant contribution (via KQ phonon side band, dark red) decreases sharply under strain, cf. (a), whereas the resonant (via KK-KQ hybridization, bright red) contribution reaches its maximum when KK and KQ excitons are energetically degenerate (at strain value of ~0.35%). Experimentally obtained X_{KQ} intensity (red squares) relative to the X_{KK}^0 intensity, measured in the center of the membrane, follows the trend of resonant brightening of KQ. Note large uncertainty (red-shaded) below 0.45% strain due to mixed X_{KK}^0 and X_{KQ}. These observations are consistent with the theoretical predictions for resonant brightening of KQ exciton and confirm that its oscillator strength is critically sensitive to the local strain value.

d) Relative KQ intensity in 1L-WSe_2 with respect to the X_{KK}^0 (red circles) vs. membrane position at $\varepsilon_{center} \approx$ 0.65%; red shadow denotes uncertainty; gray shadows correspond to the region where the edge effect becomes dominant and extracting relative intensities is challenging. The increasing KQ intensity towards x = ± 1 μm is indicative of the hybridization point shifting away from the center of the membrane. The inset shows a cartoon representation of KQ emission via resonant brightening when ε_{center} exceeds the threshold strain for KK-KQ hybridization.

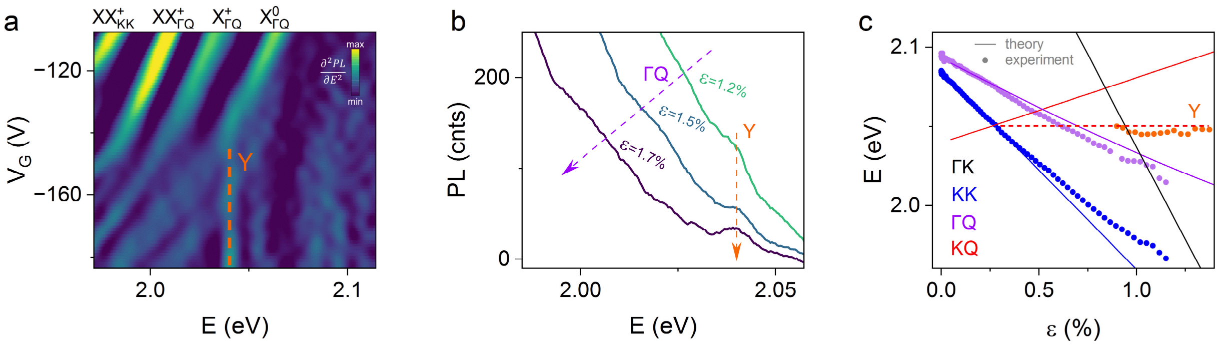

Figure S8 Strain-independent free exciton features in WS_2.

a) False color map of d^2PL/dE^2 vs. gate voltage (V_G) for the same device as in the Fig. 2 and 3 of the main text. Near V_G = -150 V, a state at 2.04 eV (orange dashed line, labelled 'Y') becomes distinguishable from the red-shifting $X_{\Gamma Q}{}^0$, and shows no strain dependence.

b) PL line cuts at 1.2%, 1.5%, and 1.7% strain. The purple arrow highlights the red shifting ΓQ peak and the orange arrow shows the Y peak becoming prominent at higher strain. This peak shows linear power dependence, supporting its free excitonic character.

c) Comparison of the experimentally obtained energy shift of $X_{KK}{}^0$, $X_{\Gamma Q}{}^0$ and Y (blue, purple, and orange, respectively) with theoretically calculated strain response of various excitons in 1L-WS_2. The dashed red line denotes the proposed KK-KQ hybridization. However, due to a relatively strong emission from $X_{\Gamma Q}{}^0$, we could not resolve the KQ exciton near 0.3% strain where the KK-KQ hybridization is theoretically predicted. The appearance of the Y peak also coincides with the strain range, where the ΓK, ΓQ, and KQ excitons are predicted to be within 50 meV energetic proximity (see Fig. S1). This may, in turn, lead to a rather complex excitonic picture and requires further investigation to assign the character of the Y peak.

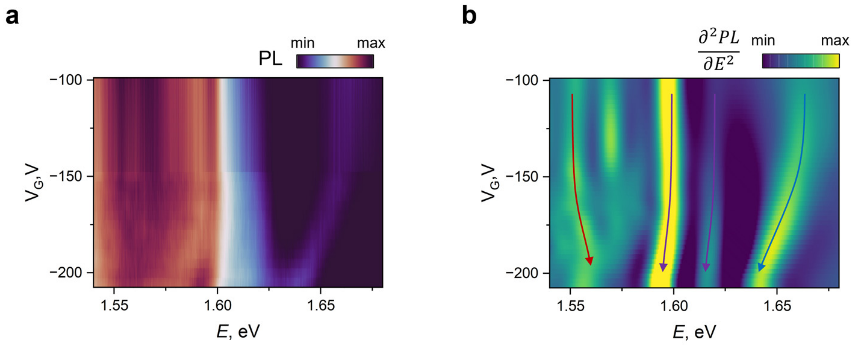

Figure S9 PL vs. strain in a bilayer WSe_2.

a) False color map of normalized PL vs. V_G in a bilayer WSe_2 at T = 10 K (same device as in Fig. 3f of the main text).

b) False color map of d^2PL/dE^2 vs V_G from the data in a). The arrows highlight peaks corresponding to KK (blue), KQ (red), and ΓQ (purple) excitons. The peak near 1.60 eV at zero strain red shifts at the one-third rate of the KK exciton (1.67 eV at zero strain). Based on this, we assign the 1.60 eV peak to the ΓQ exciton. The peak near 1.55 eV at zero strain is ~120 meV below KK excitons and blue shifts at the two-thirds of rate of KK, therefore we assign this peak to KQ exciton.

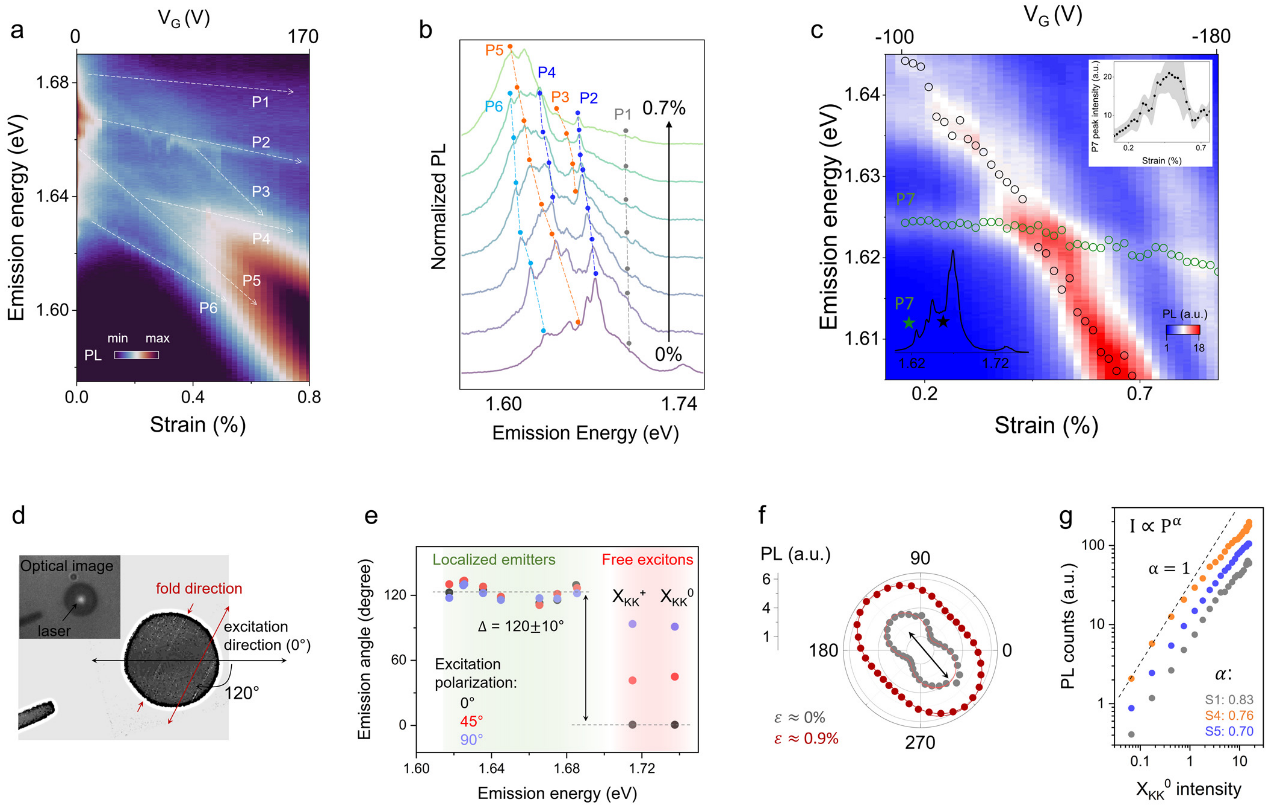

Figure S10 Detailed analysis of quantum-confined excitons in 1L-WSe_2.

a) PL vs. strain false color map in 1L-WSe_2 corresponding to the data in Fig. 4 of the main text. Several sharp peaks are observed with varying gauge factors between 10-100 meV/%.

b) PL spectra at selected strain values. Several sharp features are seen. We selectively highlight 6 features, color-coded with respect to the data in Fig. 4a in the main text. Overall, we note complex excitonic features that make it challenging to identify the energy shift of localized excitons.

c) An example of strain-controlled energy crossing between the peak near 1.625 eV (green, labelled as P7) and a free exciton (black) red shifting at a higher rate. The inset (bottom left) shows the PL line cut from the unstrained device. Note that unlike the free excitons, the intensity and strain response of these emitters is sensitive to V_G polarity. The observation of the peak P7 here corresponds to a hole-doped case, consistent with previous reports of electrically-controlled localized emitters[30]. The emission intensity of this peak increases as a result of energy crossing (top-right inset, grey shadow shows uncertainty). We suggest that strain-controlled local potential landscape influences excitonic interactions and their population dynamics. A similar observation was reported recently for localized emitters in WSe_2 [31].

d) An SEM image shows folds in this WSe_2 device (red arrows). A comparison with the optical image of the device (captured during the experiments, inset) confirms that the fold is oriented clockwise at 120 degrees with respect to the p-polarization of excitation (black arrow).

e) Emission direction of various excitons for excitation polarization direction of 0, 45, and 90 degrees (black, red, blue) with respect to the black arrow in (d). While X_{KK}^0 and X_{KK}^+ retain their polarization parallel to the excitation direction (red shadow, free excitons), the peaks below 1.7 eV (green shadow) have a preferred emission direction coinciding with the direction of the fold in (d).

f) Emission direction of the peak P7 for 0% and 0.9% strain (grey and red, respectively). We observe no change in direction of the emission. This suggests that the exciton confinement is preserved under an externally applied strain of up to 0.9%.

g) Excitation power dependence of three selected peaks (P1, P4, P5 from Fig.4a in the main text). The sublinear power dependence ($\alpha < 1$, obtained from fitting the data with power law function) of these peaks suggests their localized nature. The dashed black line corresponds to $\alpha = 1$.

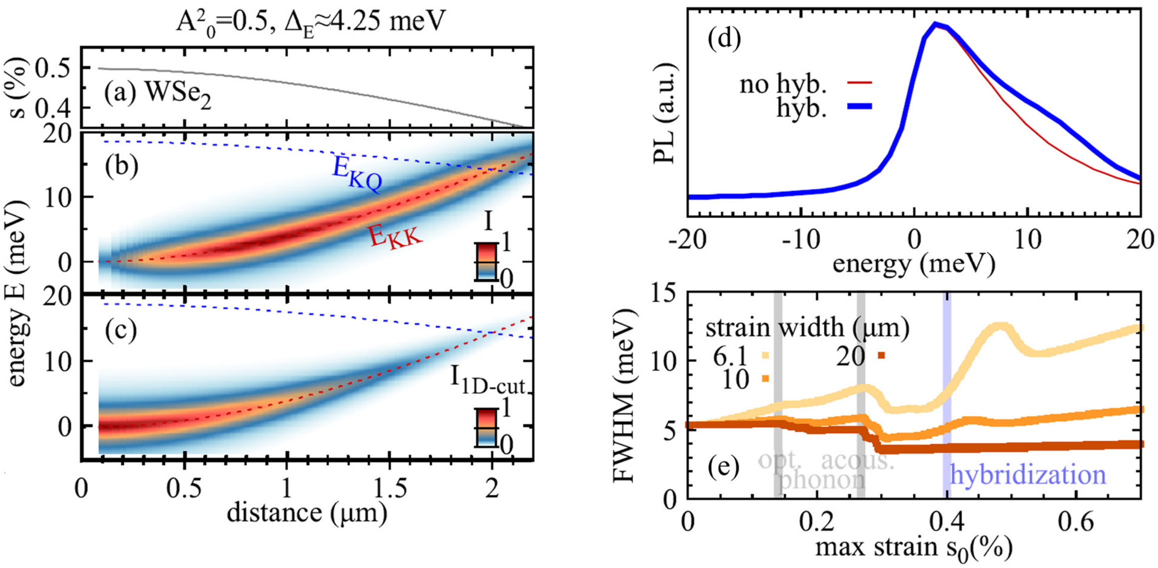

Figure S11 Effect of strain inhomogeneity on KK-KQ hybridization PL.

We analyze the impact of spatial strain inhomogeneity on the PL landscape and its effect on KK-KQ hybridization.

a) Inhomogeneous strain profile approximated via a Gaussian with FWHM 6.1 μm (obtained from the COMSOL simulations, Fig. S6).

b) Space- and energy-resolved PL, integrated over all points $\boldsymbol{r}$ equidistant from the center of the membrane (Eq. (5)). Zero energy corresponds to the energy of KK exciton at zero strain.

c) Same as in (b) but without integrating over the radial distance r. Note that only in (b) the PL has a significant value at the hybridization point (at $E_{\mathrm{KK}}^{s(r)} \approx E_{\mathrm{KQ}}^{s(r)}$, cf. the red and blue dashed lines).

d) Direct comparison of the neutral exciton PL spectra obtained from the model Note S3 with and without exciton hybridization.

e) FWHM of the space-integrated profiles (with hybridization) considering different strain width of strain distribution, showing a nearly linear increase with s_0 for widths of strain distribution up to 5 times larger than the width of exciton distribution (2 μm). Furthermore, we find step-like decreases reflecting the strain-induced closing of KK to KQ scattering channels (grey lines) as well as linewidth increases due to the hybridization (blue line).

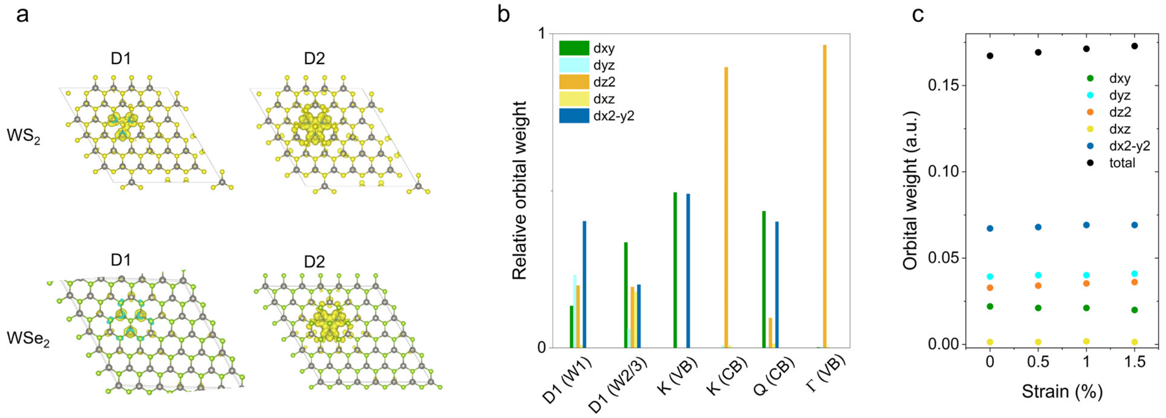

Figure S12 Extended analysis of defect exciton orbital composition.

a) Isosurface plots for the density of the defect Kohn Sham orbitals corresponding to D1 and D2 in WS_2 (top) and WSe_2 (bottom) with a single chalcogen vacancy.

b) Relative d-orbital weight at the three tungsten (W) atoms closest to the WSe_2 chalcogen vacancy defect (W1-3), compared to those of the bulk conduction and valence bands at K, Q and Γ points. The defect states are dominated by the d-orbitals of the neighbouring three W atoms (W1-3). The two W atoms W2, W3 feature almost identical orbital composition different from W1. While all five d orbitals are involved, dxy dominates for the paired W2 and W3, and dx^2-y^2 for the unpaired W1. By contrast, the CB at the K-point is dominated by dz^2 (unlike the Q-point where the orbital composition contains mainly in-plain dxy and dx^2-y^2 orbitals). Such distinct orbital composition ensures unique strain response of these valleys.

c) Strain dependence of orbital composition for D1 in 1L-WSe_2. A small change of less than 5% suggests weak dependence of orbital composition in low-strain regime used in our study.

References:

1. Rytova, N. S. *Proc. MSU, Phys., Astron.* **30**, 3 (1967).
2. Keldysh, L. Coulomb interaction in thin semiconductor and semimetal films. *JETPL* **29**, 658 (1979).
3. Brem, S. *et al.* Intrinsic lifetime of higher excitonic states in tungsten diselenide monolayers. *Nanoscale* **11**, 12381–12387 (2019).
4. Kormányos, A. *et al. kp*-theory for two-dimensional transition metal dichalcogenide semiconductors. *2D Mater.* **2**, 022001 (2015).
5. Khatibi, Z. *et al.* Impact of strain on the excitonic linewidth in transition metal dichalcogenides. *2D Mater.* **6**, 015015 (2018).
6. Zollner, K., Junior, P. E. F. & Fabian, J. Strain-tunable orbital, spin-orbit, and optical properties of monolayer transition-metal dichalcogenides. *Phys. Rev. B* **100**, 195126 (2019).
7. Kośmider, K., González, J. W. & Fernández-Rossier, J. Large spin splitting in the conduction band of transition metal dichalcogenide monolayers. *Phys. Rev. B* **88**, 245436 (2013).
8. Rasmussen, F. A. & Thygesen, K. S. Computational 2D Materials Database: Electronic Structure of Transition-Metal Dichalcogenides and Oxides. *The Journal of Physical Chemistry C* **119**, 13169–13183 (2015).
9. Hagel, J., Brem, S., Linderälv, C., Erhart, P. & Malic, E. Exciton landscape in van der Waals heterostructures. *Phys. Rev. Res.* **3**, 043217 (2021).
10. Brem, S. *et al.* Phonon-Assisted Photoluminescence from Indirect Excitons in Monolayers of Transition-Metal Dichalcogenides. *Nano Lett.* **20**, 2849–2856 (2020).
11. Deilmann, T. & Thygesen, K. S. Finite-momentum exciton landscape in mono- and bilayer transition metal dichalcogenides. *2D Mater.* **6**, 035003 (2019).
12. Blundo, E. *et al.* Strain-Induced Exciton Hybridization in WS 2 Monolayers Unveiled by Zeeman-Splitting Measurements. *Phys. Rev. Lett.* **129**, 067402 (2022).
13. Kulig, M. *et al.* Exciton Diffusion and Halo Effects in Monolayer Semiconductors. *Phys. Rev. Lett.* **120**, 207401 (2018).

14. Cadiz, F. *et al.* Exciton diffusion in WSe_2 monolayers embedded in a van der Waals heterostructure. *Appl. Phys. Lett.* **112**, 152106 (2018).

15. Selig, M. *et al.* Excitonic linewidth and coherence lifetime in monolayer transition metal dichalcogenides. *Nature Communications* **7**, (2016).

16. Brem, S., Selig, M., Berghaeuser, G. & Malic, E. Exciton Relaxation Cascade in two-dimensional Transition Metal Dichalcogenides. *Sci Rep* **8**, 8238 (2018).

17. Jin, Z., Li, X., Mullen, J. T. & Kim, K. W. Intrinsic transport properties of electrons and holes in monolayer transition-metal dichalcogenides. *Phys. Rev. B* **90**, 045422 (2014).

18. Lloyd, D. *et al.* Band Gap Engineering with Ultralarge Biaxial Strains in Suspended Monolayer MoS $_2$. *Nano Lett.* **16**, 5836–5841 (2016).

19. Falin, A. *et al.* Mechanical Properties of Atomically Thin Tungsten Dichalcogenides: WS2, WSe2, and WTe2. *ACS Nano* **15**, 2600–2610 (2021).

20. Fichter, B. Some Solutions for the Large Deflections of Uniformly Loaded Circular Membranes.

21. Mei, T., Lee, J., Xu, Y. & Feng, P. X. L. Frequency tuning of graphene nanoelectromechanical resonators via electrostatic gating. *Micromachines* **9**, (2018).

22. Rosati, R. *et al.* Dark exciton anti-funneling in atomically thin semiconductors. *Nature Communications* **12**, 7221 (2021).

23. Niehues, I. *et al.* Strain Control of Exciton–Phonon Coupling in Atomically Thin Semiconductors. *Nano Lett.* **18**, 1751–1757 (2018).

24. Aslan, B. *et al.* Excitons in strained and suspended monolayer WSe2. *2D Mater.* **9**, 015002 (2021).

25. Schmidt, R. *et al.* Reversible uniaxial strain tuning in atomically thin WSe2. *2D Mater.* **3**, 021011 (2016).

26. Blundo, E. *et al.* Evidence of the direct-to-indirect band gap transition in strained two-dimensional WS 2 , MoS 2 , and WSe 2. *Phys. Rev. Research* **2**, 012024 (2020).

27. Carrascoso, F., Li, H., Frisenda, R. & Castellanos-Gomez, A. Strain engineering in single-, bi- and tri-layer MoS2, MoSe2, WS2 and WSe2. *Nano Research* **14**, 1698–1703 (2021).

28. Zhong, Y. *et al.* A unified approach and descriptor for the thermal expansion of two-dimensional transition metal dichalcogenide monolayers. *Science Advances* **8**, eabo3783 (2022).
29. Wagner, K. *et al.* Autoionization and Dressing of Excited Excitons by Free Carriers in Monolayer WSe2. *Phys. Rev. Lett.* **125**, 267401 (2020).
30. Zipfel, J. *et al.* Electron recoil effect in electrically tunable MoSe2 monolayers. *Phys. Rev. B* **105**, 075311 (2022).
31. Electrically driven strain-induced deterministic single-photon emitters in a van der Waals heterostructure | Science Advances. https://www.science.org/doi/10.1126/sciadv.abj3176.
32. Savaresi, M. *et al.* Strain-induced dynamic control over the population of quantum emitters in two-dimensional materials. Preprint at https://doi.org/10.48550/arXiv.2301.10273 (2023).
